# Activation of Toll-Like Receptor 9 Impairs Blood Flow Recovery After Hind-Limb Ischemia

**DOI:** 10.3389/fcvm.2018.00144

**Published:** 2018-10-16

**Authors:** Sachiko Nishimoto, Kunduziayi Aini, Daiju Fukuda, Yasutomi Higashikuni, Kimie Tanaka, Yoichiro Hirata, Shusuke Yagi, Kenya Kusunose, Hirotsugu Yamada, Takeshi Soeki, Michio Shimabukuro, Masataka Sata

**Affiliations:** ^1^Department of Cardiovascular Medicine, Tokushima University Graduate School of Biomedical Sciences, Tokushima, Japan; ^2^Department of Cardio-Diabetes Medicine, Tokushima University Graduate School of Biomedical Sciences, Tokushima, Japan; ^3^Department of Cardiovascular Medicine, The University of Tokyo, Tokyo, Japan; ^4^Division for Health Service Promotion, The University of Tokyo, Tokyo, Japan; ^5^Department of Pediatrics, The University of Tokyo Hospital, Tokyo, Japan; ^6^Department of Diabetes, Endocrinology and Metabolism, School of Medicine, Fukushima Medical University, Fukushima, Japan

**Keywords:** hind-limb ischemia, blood flow recovery, Toll-like receptor 9, inflammation, macrophage

## Abstract

**Background:** Peripheral artery disease causes significant functional disability and results in impaired quality of life. Ischemic tissue injury releases various endogenous ligands for Toll-like receptors (TLRs), suggesting the involvement of TLRs in blood flow recovery. However, the role of TLR9, which was originally known as a sensor for bacterial DNA, remains unknown. This study investigated the role of TLR9 in blood flow recovery in the ischemic limb using a mouse hind-limb ischemia model.

**Methods and Results:** Unilateral femoral artery ligation was performed in TLR9-deficient (*Tlr9*^−/−^) mice and wild-type mice. In wild-type mice, femoral artery ligation significantly increased mRNA expression of TLR9 in the ischemic limb (*P* < 0.001) and plasma levels of cell-free DNA (cfDNA) as determined by single-stranded DNA (ssDNA) (*P* < 0.05) and double-stranded DNA (dsDNA) (*P* < 0.01), which are endogenous ligands for TLR9, compared with the sham-operated group. Laser Doppler perfusion imaging demonstrated significantly improved ratio of blood flow in the ischemic to non-ischemic limb in *Tlr9*^−/−^ mice compared with wild-type mice at 2 weeks after ligation (*P* < 0.05). *Tlr9*^−/−^ mice showed increased capillary density and reduced macrophage infiltration in ischemic limb. Genetic deletion of TLR9 reduced the expression of TNF-α, and attenuated NF-κB activation in ischemic muscle compared with wild-type mice (*P* < 0.05, respectively) at 3 days after the surgery. ODN1826, a synthetic agonistic oligonucleotide for TLR9, or plasma obtained from mice with ischemic muscle promoted the expression of TNF-α in wild-type macrophages (*P* < 0.05), but not in *Tlr9*^−/−^ macrophages. ODN1826 also activated NF-κB signaling as determined by the degradation of IκBα in wild-type macrophages (*P* < 0.05), but not in *Tlr9*^−/−^ macrophages. In vitro experiments using human umbilical vein endothelial cells demonstrated that TNF-α, or conditioned medium obtained from wild-type macrophages treated with ODN1826 accelerated cell death as determined by MTS assay (*P* < 0.05 and *P* < 0.01, respectively).

**Conclusion:** Our results suggest that ischemic muscle releases cfDNA, which activates TLR9 and enhances inflammation, leading to impairment of blood flow recovery in the ischemic limb. cfDNA-TLR9 signaling may serve as a potential therapeutic target in ischemic limb disease.

## Introduction

Peripheral artery disease (PAD), due to partial or complete obstruction of the arteries, is one of the most common manifestations of atherosclerosis, which is associated with increased risk of cardiovascular events (1). Collateral vessel development is a physiological response in ischemic tissues to compensate reduced blood flow. Previous studies indicated that an inflammatory process regulates neovascularization and subsequent blood flow recovery (2). In this process, the immune system plays a pivotal role (3). Exogenous ligands such as microbial-associated molecules activate macrophages through Toll-like receptors (TLRs), whereas endogenous molecules derived from damaged and dead host cells also activate macrophages though TLRs and promote sterile inflammation by inducing cytokines (4). Ischemia induces cellular and tissue damage (5), causing the release of cellular debris which contains various endogenous ligands for TLRs. In fact, several studies reported that TLR-2, 3 and 4 participate in blood flow recovery in ischemic tissues by modulating inflammation and angiogenesis (6–11). However, the role of TLR9 in blood flow recovery in ischemic tissues remains unknown. TLR9 recognizes exogenous DNA fragments which contain unmethylated CpG-DNA and plays a role in the innate immune system (12, 13). In addition, accumulating evidence has revealed that TLR9 has a broad range of functions in pathophysiological conditions. TLR9 recognizes cell-free DNA (cfDNA) released from degenerated tissues/cells and activates inflammatory cells including macrophages, leading to the development of sterile inflammation-associated diseases such as autoimmune diseases, insulin resistance, and others (14–16).

Therefore, in this study, we hypothesized that TLR9 activation by cfDNA released from ischemic tissues promotes inflammation and modulates blood flow recovery in the ischemic limb. To test our hypothesis, we induced hind-limb ischemia in wild-type and TLR9-deficient (*Tlr9*^−/−^) mice and compared blood flow recovery and the degree of inflammation in the ischemic limb between these strains of mice. Also, we investigated the role of TLR9 in the link between inflammation and blood flow recovery in ischemic tissue.

## Materials and methods

### Animals

C57BL/6 (wild-type) mice and *Tlr9*^−/−^ mice (C57BL/6 background) were obtained from Japan SLC Inc. and Oriental BioService, Inc., respectively. Mice were maintained under a 12-h light/dark cycle, with a standard diet and water *ad libitum*. All experimental procedures conformed to the guidelines for animal experimentation of Tokushima University. The protocol was reviewed and approved by our institutional ethics committee under No. T29-96.

### Hind-limb ischemia by ligation of femoral artery and blood flow monitoring

Unilateral hind-limb ischemia was induced in wild-type mice and *Tlr9*^−/−^ mice as described previously (17). Briefly, the animals were anesthetized by intraperitoneal injection of pentobarbital (50 mg/kg). The proximal and distal portions of the femoral artery and the distal portion of the saphenous artery were ligated. The arteries and all side branches were dissected free and excised. Blood flow in limbs was measured using a laser Doppler blood perfusion monitor (Moor LDPI) pre- and post-operatively and on days 7 and 14. Blood flow recovery in the ischemic limb was determined by the ratio of perfusion in the ischemic limb to the untreated limb.

### Immunohistochemical staining

Capillary density in the ischemic limb was measured as described previously (17). At 14 days after femoral artery ligation, mice were sacrificed by intraperitoneal injection of an overdose of pentobarbital. The whole limbs were fixed in methanol overnight, and the ischemic muscles were embedded in paraffin. Sections (5 μm) were de-paraffinized and incubated with an anti-CD31 antibody (clone MEC13.1, BD Pharmingen). Antibody distribution was detected with the avidinbiotin-complex technique and a Vector Red Alkaline Phosphatase substrate kit (Vector Laboratories). Nuclei were stained with hematoxylin. Six different fields from each section were randomly selected, and visible CD31-positive capillaries were quantitated with a FLOVEL Filing System (FLOVEL Company, Ltd.). Capillary density was expressed as the number of capillaries per field or per muscle fiber.

Accumulation of macrophages in ischemic limb was also examined by immunohistochemistry. Sections were incubated with anti-Mac3 antibody (BD Pharmingen). Development was performed by the combination of HRP-conjugated secondary antibody and ImmPACT DAB substrate (Vector Laboratories). Sections were counterstained with hematoxylin. Four different fields from each section were randomly selected, and Mac3-positive cells were quantitated with CellSens (OLYMPUS). Accumulation of macrophages was determined as the number of macrophages per field or per muscle fiber.

### Cell culture

Thioglycollate-stimulated peritoneal macrophages were collected with cold PBS from age-matched female wild-type mice or *Tlr9*^−/−^ mice at the age of 8-12 weeks and cultured in DMEM containing 10% FBS at 37°C in a humidified incubator in 5% CO_2_ and 95% air. At 24 h after plating, isolated peritoneal macrophages were used for experiments. Peritoneal macrophages were stimulated with ODN1826 or control-ODN1826 (GeneDesign, Inc.), synthetic oligonucleotides that contain unmethylated CpG, for indicated time. We collected plasma from wild-type mice at 3 days after femoral artery ligation or sham-operation. Wild-type or *Tlr9*^−/−^ macrophages were stimulated with these plasma for 4 h.

Human umbilical vein endothelial cells (HUVEC) were purchased from Life Technologies and grown in EGM-2 (Lonza) at 37°C in a humidified incubator in 5% CO_2_ and 95% air. HUVEC (passages 4-6) were used for experiments.

### Assessment of cell viability

HUVEC were plated in 96-well plates and grown to 40~50% confluence in EGM-2, and then stimulated with or without human recombinant TNF-α for 24 h in EBM-2 containing 1% FBS. Cell viability of HUVEC was determined by MTS assay using a CellTiter 96 AQueous One Solution Cell Proliferation Assay kit (Promega) according to the manufacturer's instructions. The percentage of absorbance was calculated against that of non-stimulated cells. Cell viability of HUVEC was also examined after treatment with conditioned medium (CM) of macrophages. Wild-type macrophages or *Tlr9*^−/−^ macrophages were pretreated with ODN1826 or control-ODN1826 (100 nM) under serum-starved conditions for 24 h, and then cells were cultured for another 24 h in a starvation medium without synthetic oligonucleotides. CM was collected after centrifugation and filtration through a 40-μm mesh to remove cell debris. Cell viability was determined by MTS assay after 72-h incubation.

### Measurement of cfDNA in plasma

cfDNA in plasma was extracted using a QIAamp DNA Mini Kit (Qiagen), according to the manufacturer's instructions. The concentrations of single-stranded DNA (ssDNA) and double-stranded DNA (dsDNA) in extracted cfDNA were measured using a QuantiFluor ssDNA System (Promega) and Quant-iT PicoGreen dsDNA Assay Kit (Life Technologies), respectively, according to the instructions.

### Reverse transcription and quantitative real-time polymerase chain reaction

RNA extracted from muscles and cells with an illustra RNAspin RNA Isolation Kit (GE Healthcare) was used for cDNA synthesis using a QuantiTect Reverse Transcription kit (Qiagen). Quantitative real-time RT-PCR (qPCR) was performed using Power SYBR Green PCR Master Mix (Applied Biosystems) and gene-specific primers on an Mx3000P (Agilent Technologies). Data are expressed in arbitrary units normalized by β-actin. The sequences of primers used in this study were as follows: TNF-α, sense 5′-accctcacactcagatcatcttc-3′ and antisense 5′-tggtggtttgctacgacgt-3′; F4/80, sense 5′-tgcatctagcaatggacagc-3′ and antisense 5′-gccttctggatcCATttgaa-3′; β-actin, sense 5′-CCTgagcgcaagtactctgtgt-3′ and antisense 5′-gctgatccacatctgctggaa-3′.

### Western blot analysis

Protein lysates were isolated from tissues or cells using RIPA buffer (Wako Pure Chemical Industries, Ltd.) containing a protease inhibitor cocktail (Takara Bio Inc.) and phosphatase inhibitors (Roche LifeScience). Proteins were separated by SDS-PAGE and transferred to polyvinilidine difluoride membranes (Hybond-P; GE Healthcare). The membrane was blocked in 5% BSA for 1 h at room temperature, followed by incubation with a primary antibody against either total-IκBα (Cell Signaling Technology), TNF-α (abcam), α-Tubulin (MBL), or β-actin (Sigma) at 4°C overnight. After blots were washed in TBS containing 1% Tween-20, the membranes were incubated in horseradish peroxidase-conjugated secondary antibody (Chemicon) for 1 h. Expression of α-Tubulin or β-actin was used as an internal control to confirm equivalent total protein loading. Antibody distribution was visualized with ECL-plus reagent (GE Healthcare) using a luminescent image analyzer (LAS-1000, Fuji Film).

### Statistical analysis

Data were expressed as mean ± SEM. Comparisons between two groups were made by unpaired Student's *t* test. Comparisons of multiple groups were made by one-way analysis of variance (ANOVA) followed by Tukey test. *P* < 0.05 was considered statistically significant.

## Results

### Femoral artery ligation increased expression of TLR9 in ischemic limb and endogenous ligand for TLR9 in blood

**Figure 1:**
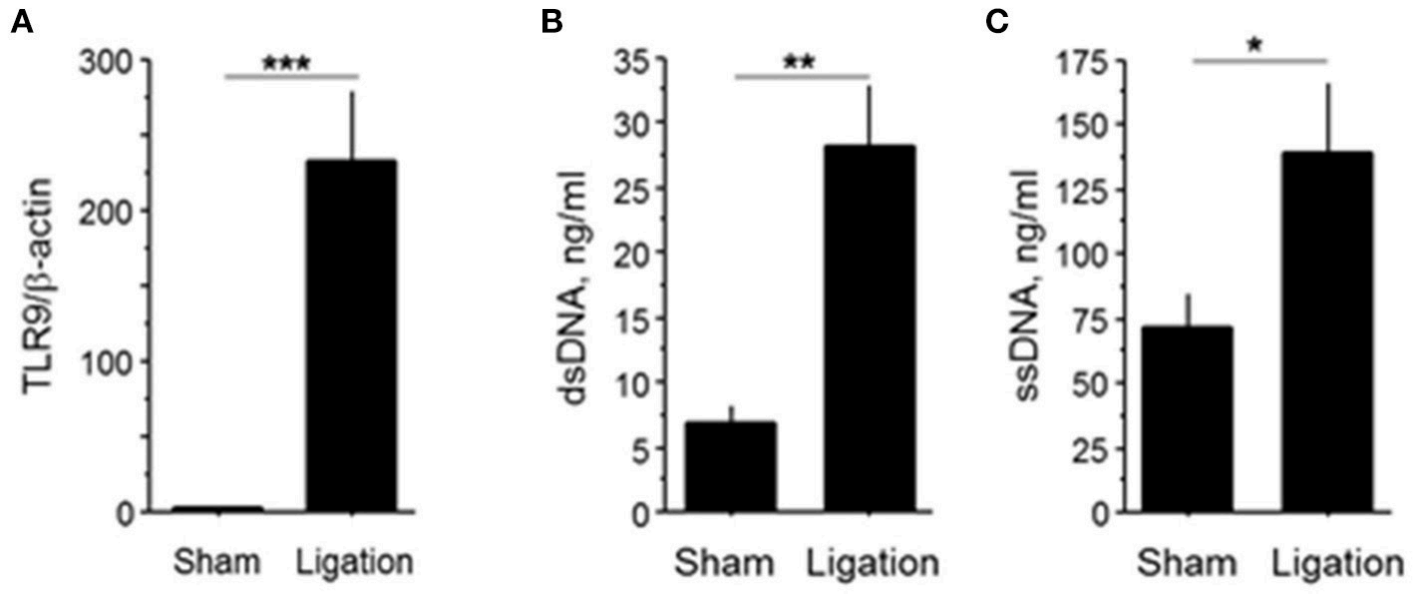
Femoral artery ligation increased TLR9 expression in ischemic limb and circulating level of endogenous TLR9 ligand. **(A)** Femoral artery ligation increased TLR9 expression in the ischemic limb at 3 days after surgery in wild-type mice (*N* = 7, per group). **(B,C)** Induction of ischemia increased plasma circulating level of cfDNA as determined by dsDNA and ssDNA at 3 days after surgery in wild-type mice (*N* = 7, ligated group; *N* = 8, sham operated group). ^*^P < 0.05, ^**^P < 0.01, and ^***^P < 0.001. All values are mean ± SEM.

To determine the participation of TLR9 in blood flow recovery of the ischemic limb, we examined the expression of TLR9 and circulating level of TLR9 ligand at 3 days after femoral artery ligation in wild-type mice. Results of qPCR demonstrated that ligation of the femoral artery markedly increased *Tlr9* expression in the ischemic limb compared to the sham operated group (Figure 1A). Induction of hind-limb ischemia in wild-type mice also increased the plasma levels of dsDNA and ssDNA, internal ligands for TLR9, compared with the sham-operated group (Figures 1B,C).

### TLR9 deficiency increased blood flow recovery and capillary density in ischemic limb

**Figure 2:**
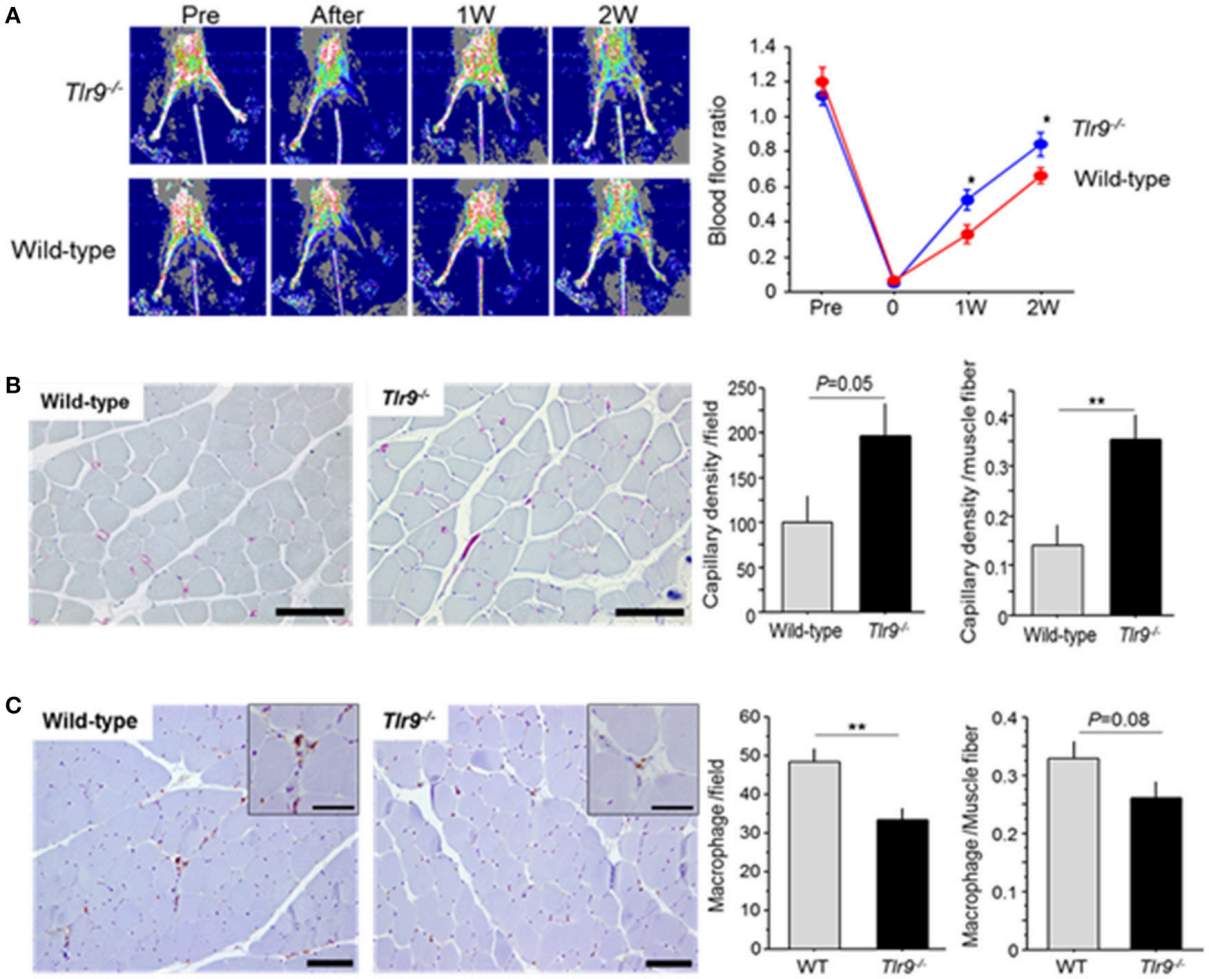
TLR9 deficiency increased blood flow recovery and reduced macrophage infiltration in ischemic limb. **(A)** Wild-type mice and *Tlr9*^−/−^ mice were subjected to hind-limb ligation surgery, and serial blood flow in the ischemic limb was monitored. In laser Doppler perfusion images, blue color represents lower perfusion and red color represents higher reperfusion. The ratio of flow (ischemic limb/non-ischemic limb) was quantitated. *Tlr9*^−/−^ mice showed better blood flow recovery compared with wild-type mice (*N* = 17, per group). **(B)** Capillary density in gastrocnemius muscle was determined by immunohistochemical staining. Representative images of CD31 staining (red; CD31 positive) are shown. Capillary density was higher in *Tlr9*^−/−^ mice compared with wild-type mice (*N* = 9, per group). Bar; 100 μm. **(C)** Infiltration of macrophages into the ischemic limb was examined by immunohistochemistry. *Tlr9*^−/−^ mice showed reduced macrophage infiltration as determined by Mac3 staining at 14 days after surgery compared with wild-type mice. (*N* = 9, per group). Bar; 100 μm. Insets are larger magnification (Bar; 50 μm). ^*^P < 0.05 and ^**^P < 0.01. All values are mean ± SEM.

Wild-type mice and *Tlr9*^−/−^ mice were subjected to femoral artery ligation, and serial blood flow measurements were performed by laser Doppler imaging. *Tlr9*^−/−^ mice showed better blood flow recovery than wild-type mice (Figure 2A). To clarify the role of TLR9 in the process of blood flow recovery, we examined capillary density in the ischemic limb by histological analysis. The results of CD31 staining showed that capillary density per square meter or per muscle fiber in the ischemic limb in *Tlr9*^−/−^ mice was higher than that in wild-type mice at 14 days after ligation (Figure 2B). We also found that *Tlr9*^−/−^ mice showed reduced infiltration of macrophages, one of the key players in the development of inflammation in the ischemic limb at 14 days after surgery, compared with wild-type mice (Figure 2C).

### TLR9 deficiency reduced inflammatory response in ischemic limb

**Figure 3:**
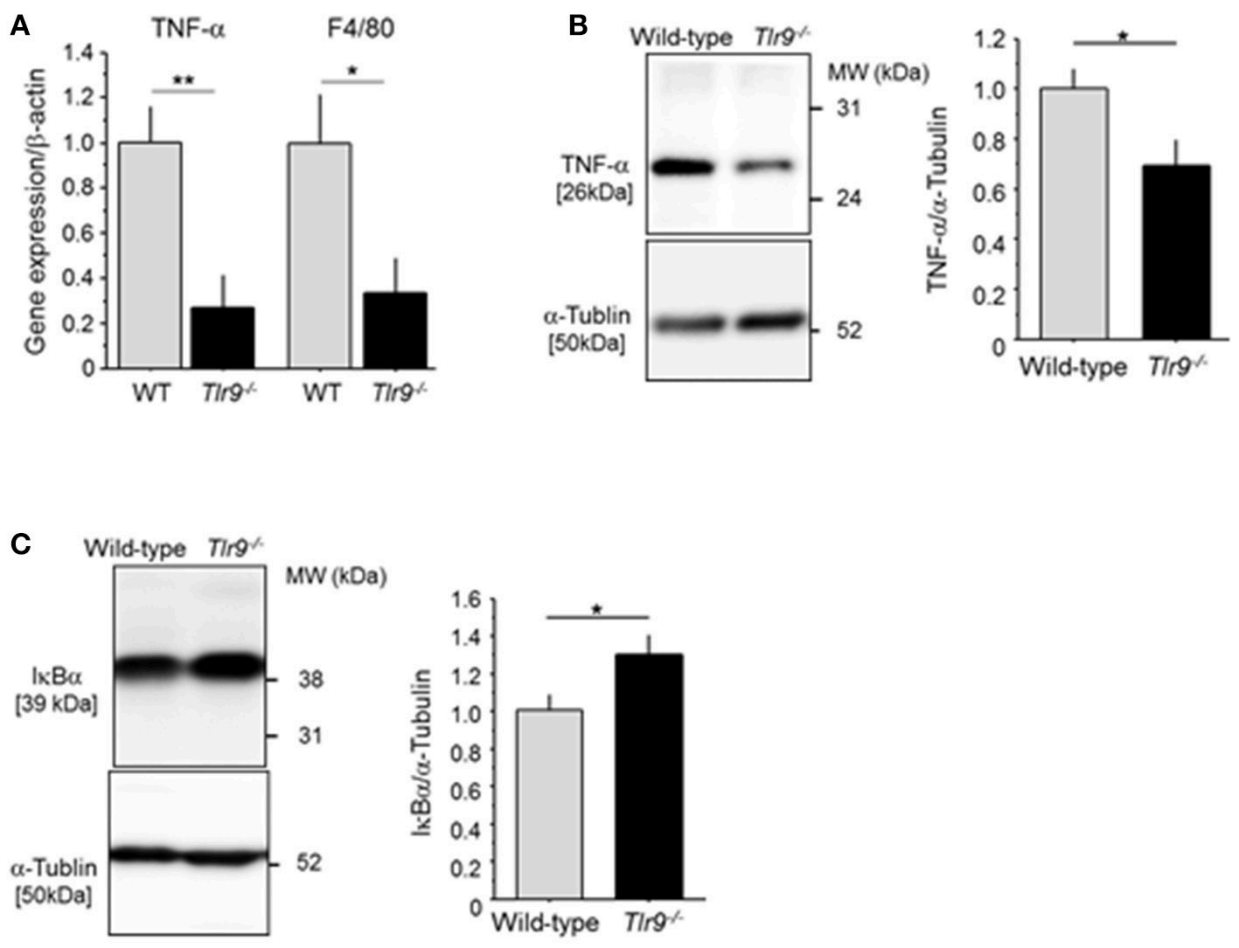
TLR9 deficiency reduced inflammation in ischemic limb. **(A)** The result of qPCR analysis demonstrated that the expression of TNF-α and F4/80 in ischemic limb at 3 days after surgery was lower in *Tlr9*^−/−^ mice than in wild-type mice (*N* = 6-7, per group). **(B)** Protein level of TNF-α in ischemic muscle in *Tlr9*^−/−^ mice was lower than that in wild-type mice (*N* = 4-6, per group). **(C)** The degradation of IκBα in the ischemic limb was significantly suppressed in *Tlr9*^−/−^ mice compared with wild-type mice, suggesting suppression of NF-κB signaling in *Tlr9*^−/−^ mice (*N* = 4-6, per group). ^*^P < 0.05 and ^**^P < 0.01. All values are mean ± SEM.

We examined the effect of TLR9 deficiency on the inflammatory process in the ischemic limb. The results of qPCR demonstrated that *Tlr9*^−/−^ mice had reduced expression of TNF-α and F4/80, a macrophage marker, in the ischemic limb at 3 days after surgery compared with wild-type mice (Figure 3A). The results of western blotting confirmed reduced expression of TNF-α in *Tlr9*^−/−^ mice at the protein level (Figure 3B). In addition, the results of western blotting using ischemic limbs showed that TLR9 deficiency significantly suppressed the degradation of IκBα compared with wild-type mice, indicating reduced activation of NF-κB signaling, one of the regulator TNF-α expression, in the ischemic limb in *Tlr9*^−/−^ mice (Figure 3C).

### Activation of TLR9 promoted expression of TNF-α in macrophages

**Figure 4:**
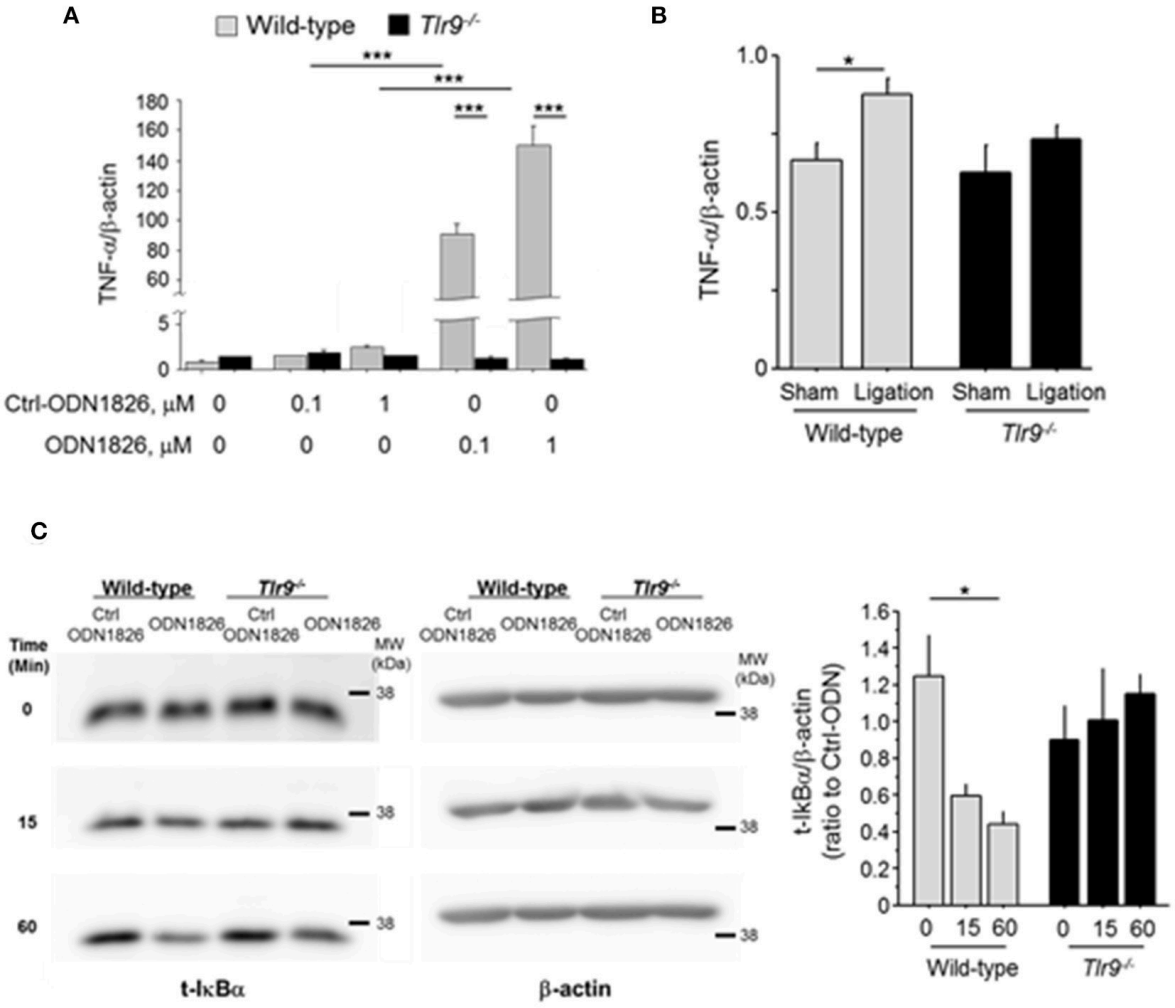
TLR9 activation promoted TNF-α expression in macrophages. **(A)** Activation of TLR9 by ODN1826 (0.1-1.0 μM) for 4 h promoted the expression of TNF-α in wild-type macrophages but not in *Tlr9*^−/−^ macrophages (*N* = 4, per group). **(B)** Wild-type or *Tlr9*^−/−^ macrophages were treated with plasma obtained from wild-type mice which received femoral ligation or sham operation for 4 h. Plasma from mice with ischemic muscle increased TNF-α expression in wild-type macrophages compared with that from sham-operated mice, however, plasma from mice with ischemic muscle did not show this response in *Tlr9*^−/−^ macrophages (*N* = 4-6, per group). **(C)** ODN1826 promoted degradation of IκBα in wild-type macrophages in time dependent manner, suggesting the activation of NF-κB signaling. In *Tlr9*^−/−^ macrophages, this response was not observed. ^*^P < 0.05 and ^***^P < 0.001. All values are mean ± SEM.

Inflammation associated with macrophage activation plays a pivotal role in blood flow recovery in the ischemic limb. Therefore, we examined the effect of TLR9 activation in macrophages. The results of *in vitro* experiments using peritoneal macrophages demonstrated that TLR9 activation by ODN1826, a synthetic oligonucleotide which activates TLR9, promoted the expression of TNF-α in wild-type macrophages, but not in *Tlr9*^−/−^ macrophages (Figure 4A). We further stimulated these macrophages with plasma obtained from mice which received femoral artery ligation or sham-operation. Wild-type macrophages increased TNF-α expression in the presence of plasma from mice with ischemic muscle compared with that from sham-operated mice, however, *Tlr9*^−/−^ macrophages did not show this response (Figure 4B). The ligation of TLR9 agonist to wild-type macrophages significantly activated NF-κB signaling as determined by the degradation of IκBα, however, this response was not observed in *Tlr9*^−/−^ macrophages (Figure 4C).

### TLR9-mediated macrophage activation attenuated cell viability of endothelial cells

**Figure 5:**
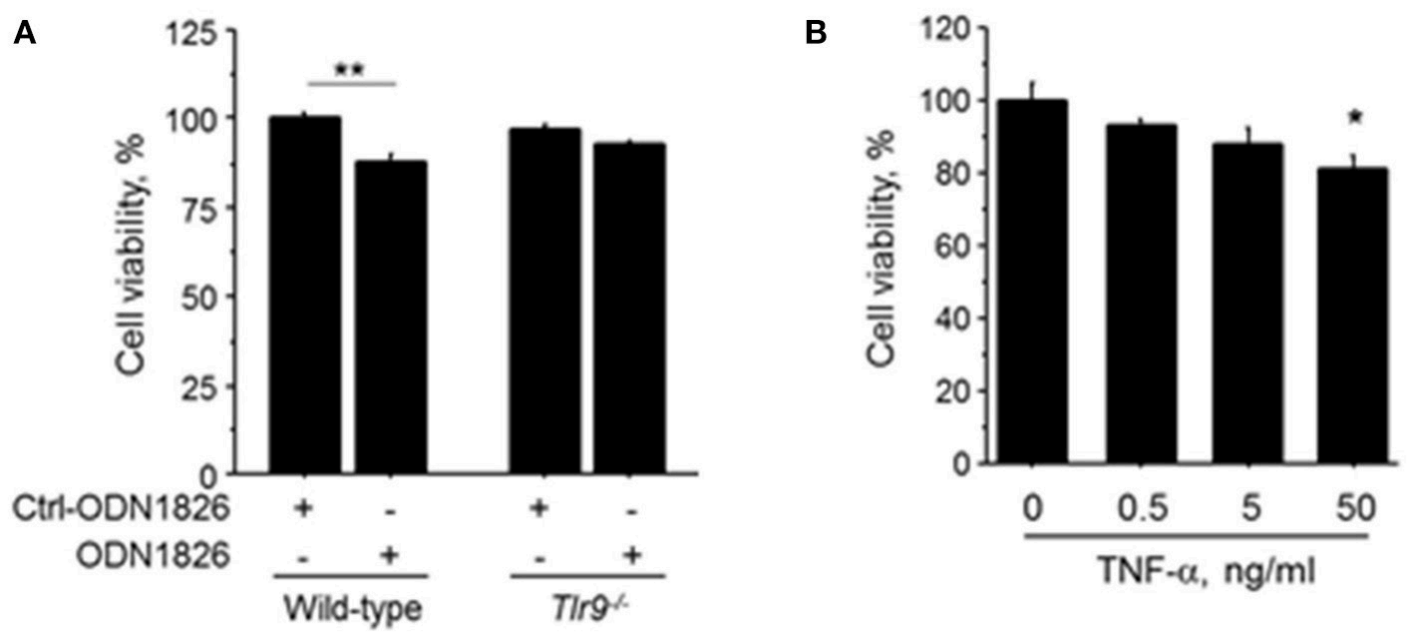
TLR9-induced macrophage activation accelerated cell death of HUVEC. **(A)** HUVEC were treated with the CM obtained from wild-type or *Tlr9*^−/−^ macrophages treated with ODN1826 or control-ODN1826. At 72 h after treatment, the viability of HUVEC determined by MTS assay was significantly reduced by CM obtained from wild-type macrophage activated by ODN1826, although the viability of HUVEC was not affected by CM obtained from *Tlr9*^−/−^ macrophage (*N* = 5, per group). **(B)** Stimulation with TNF-α for 24 h decreased cell viability in HUVEC as determined by MTS assay (*N* = 4-6, per group). ^*^P < 0.05 and ^**^P < 0.01. All values are mean ± SEM.

To investigate the effect of TLR9-mediated macrophage activation on endothelial cell, we treated HUVEC with the CM obtained from wild-type macrophages or *Tlr9*^−/−^ macrophages treated with ODN1826. CM obtained from wild-type macrophage treated with a TLR9 agonist significantly reduced the viability of HUVEC determined by MTS assay, although CM from *Tlr9*^−/−^ macrophage did not have any effect (Figure 5A). HUVEC treated with TNF-α also showed attenuated cell viability (Figure 5B). These results suggest that TNF-α, produced by macrophages via TLR9 activation, accelerated cell death of endothelial cells, leading to the suppression of blood flow recovery in ischemic limb.

## Discussion

In this study, we demonstrated that femoral artery ligation increased TLR9 expression in the ischemic limb and circulating cfDNA, an endogenous ligand for TLR9, in plasma. The results of *in vivo* experiment demonstrated that genetic deletion of TLR9 increased capillary density in ischemic muscle and promoted blood flow recovery. Genetic deletion of TLR9 attenuated inflammation as determined by TNF-α expression in the ischemic limb. The results of *in vitro* experiments using macrophages and HUVEC indicated that TLR9-mediated macrophage activation attenuated cell viability of HUVEC. These results suggest that TLR9 activation caused by cfDNA released from ischemic muscle promotes inflammation, leading to deterioration of blood flow recovery.

Accumulating evidence has suggested the involvement of the innate immune system in the pathophysiology of various diseases (18–21). In this process, host-derived internal ligands initiate and promote sterile chronic inflammation. Ischemic or damaged tissue releases various molecules, such as fatty acids, nuclear proteins, and nucleic acids, which can activate pattern recognition receptors including TLRs as endogenous ligands (22). cfDNA is one of these internal ligands which activates TLR9. Previous studies have reported the association of circulating cfDNA level with the pathophysiology and/or severity of chronic inflammatory diseases, including severe cardiovascular diseases, and suggested the role of cfDNA as a biomarker of several chronic inflammatory diseases (16, 23, 24). Our results showed that hind-limb ischemia increased TLR9 expression in ischemic muscle and the circulating level of cfDNA, suggesting that cfDNA-TLR9 signaling participates in the process of blood flow recovery.

A number of previous studies reported the contribution of TLR signaling to neovascularization or blood flow recovery after ischemia. However, its roles are still under debate. TLR4 is one of the most studied TLRs from the perspective of blood flow recovery after ischemia. Several studies reported that the absence of TLR4 attenuates collateral formation and tissue perfusion after arterial occlusion (10, 25). In addition, lipopolysaccharide, an exogenous TLR4 ligand, stimulates collateral artery formation after arterial occlusion (11, 26). These studies indicated that TLR4-mediated inflammation resulted in proliferation of collateral arteries and improvement of blood flow recovery. On the contrary, recent studies demonstrated that activation of TLR4 impairs blood flow recovery after ischemia. Suppression of TLR4-mediated inflammation is associated with muscle recovery and rapid blood flow recovery in TLR4-deficient mice (11, 27). Also, lack of CD180, a specific inhibitor of the TLR4-mediated inflammatory response, accelerated inflammation and retarded blood flow recovery after ischemia (6, 28). These studies indicated that unrestrained inflammation impaired blood flow recovery. Therefore, adequate inflammation is important for promoting blood flow recovery in ischemic tissues.

Few studies have reported the role of TLR9 signaling in blood flow recovery in the ischemic limb, although several previous studies examined its role in angiogenesis using different models. One study reported that TLR9 signaling stimulated wound healing by enhancing blood flow (29). On the other hand, other studies demonstrated that TLR9 activation reduced vessel sprouting from aortic rings and hemangiogenesis and lymphangiogenesis in a suture-induced corneal angiogenesis model (30). Also, another *in vitro* study reported that TLR9 activation with a lower concentration of ODN1826, a TLR9 agonist, stimulated angiogenesis, although activation with a higher concentration of ODN1826 impaired angiogenesis (31). This study suggested dual roles of ODN1826 in angiogenesis. This discrepancy might have been due to the difference in models used in these studies; however, adequate control of TLR9 activation seems to be essential for angiogenesis.

In our present study, we demonstrated that genetic deletion of TLR9 reduced TNF-α expression in the ischemic limb. We also found that TLR9 activation by ODN1826 promoted TNF-α expression in macrophages, one of the key players in blood flow recovery in the ischemic limb. Furthermore, plasma obtained from mice with ischemic muscle increased TNF-α expression in wild-type macrophages but not in *Tlr9*^−/−^ macrophages. These results suggested that TLR9 activation by endogenous ligands released from ischemic muscle promotes TNF-α expression in macrophages, regulating blood flow recovery in ischemic limb. In this study, genetic deletion of TLR9 reduced the degradation of IκBα in ischemic muscle, suggesting suppression of NF-κB signaling. Previous studies have reported that TLR signaling pathways activate NF-κB, which regulates the expression of inflammatory cytokines including TNF-α (32, 33). As well as other TLRs, TLR9 signaling also activates this pathway (16). In fact, we demonstrated that the ligation of TLR9 agonist activates NF-κB signaling in wild-type macrophages as determined by the degradation of IκBα, but not in by *Tlr9*^−/−^ macrophages in this study. Our results suggested the link between TLR9 and NF-κB, at least partially, although further studies are needed to elucidate precise signaling in ischemic muscle. Previous studies reported that TNF-α has two contradictory functions on endothelial cells (34–36). This contradiction has been partially explained by tissue TNF-α concentration (34). That is, a low TNF-α concentration promotes, but a high TNF-α concentration inhibits angiogenesis. Previous *in vitro* studies, a higher concentration of TNF-α reduced cell viability of endothelial cells (34, 36). The result of our experiment is consistent with these studies. Furthermore, in this study, CM obtained from wild-type macrophages activated by a TLR9 agonist attenuated cell viability of HUVEC, although CM from *Tlr9*^−/−^ macrophage did not have any effect. These results suggest that suppression of TNF-α expression in TLR9-deficient macrophages is associated with higher capillary density and better blood flow recovery through inhibition of endothelial cell death. Combined with the results of previous studies, the results of our study suggest that well-controlled activation of TLR9 is important for blood flow recovery in the ischemic limb by regulating TNF-α expression. Several clinical studies have demonstrated elevated plasma level of TNF-α in patients with PAD (37, 38). Further studies are required to determine the role of cfDNA-TLR9 signaling in inflammation and blood flow recovery in ischemic tissues, which might provide a novel therapeutic strategy for PAD.

There are several limitations in this study. The time point we examined blood flow, capillary density, and inflammatory status in the ischemic limb was limited. *In vitro* experiments were also performed at limited time point. Other cell types or signaling pathways might also have roles in different time points. Second, we focused on pro-inflammatory activation of macrophages via TLR9, however, we did not perform macrophage-specific investigation in our *in vivo* studies. Therefore, other cell-types might also contribute to the results. Last, the results of our *in vivo* study suggested that TLR9-deletion promotes blood flow recovery in ischemic muscle. However, functional analysis was not performed in this study.

Inflammation regulates blood flow recovery in ischemic tissues. The results of our present study suggested that TLR9-deletion attenuates inflammatory responses and promotes blood flow recovery in ischemic muscle. These results indicate that strict control of inflammation and appropriate expression of TNF-α through TLR9 signaling in ischemic tissues are critical for blood flow recovery. Regulation of cfDNA-TLR9 signaling could be a potential therapeutic strategy for regulation and acceleration of blood flow recovery in ischemic limb injury.

## Author contributions

SN, KA, and DF designed and performed the experiments, interpreted the results, and prepared the manuscript. YaH, KT, YoH, SY, and TS assisted with *in vivo* experiments. KK, HY, and MiS contributed to data interpretation and critical reading of the manuscript. MaS interpreted the data and prepared the manuscript. All authors discussed the results and commented on the manuscript.

### Conflict of interest statement

The Department of Cardio-Diabetes Medicine, Tokushima University Graduate School, is supported in part by unrestricted research grants from Boehringer Ingelheim, Tanabe-Mitsubishi, Kowa, and Actelion. The authors declare that the research was conducted in the absence of any commercial or financial relationships that could be construed as a potential conflict of interest.

## Abbreviations

cfDNA: cell-free DNA
CM: conditioned medium
dsDNA: double-stranded DNA
HUVEC: human umbilical vein endothelial cells
PAD: peripheral artery disease
qPCR: Quantitative real-time RT-PCR
ssDNA: single-stranded DNA
TLR: Toll-like receptor
*Tlr9*^−/−^: TLR9-deficient.
